# MicroRNA-7 directly targets insulin-like growth factor 1 receptor to inhibit cellular growth and glucose metabolism in gliomas

**DOI:** 10.1186/s13000-014-0211-y

**Published:** 2014-11-14

**Authors:** Bo Wang, Fei Sun, Nan Dong, Zhenguo Sun, Yi Diao, Cheng Zheng, Jianxin Sun, Yang Yang, Dehua Jiang

**Affiliations:** Department of Neurosurgery, Xuzhou Central Hospital, Xuzhou, 221009 China

**Keywords:** Glioblastoma multiforme, miR-7, IGF-1R, AKT

## Abstract

**Background:**

Recent studies observed that altered energy metabolism has become widespread in cancer cells along with other cancer-associated traits that have been accepted as hallmarks of cancer. Akt signaling pathway is involved in the aerobic glycolysis program. However, mechanisms underlying the regulation of aerobic glycolysis and Akt activity in gliomas remain unclear. MicroRNAs are a group of small non-coding RNAs that can function as endogenous RNA interference to regulate expression of targeted genes. This study was conducted to detect the function of miR-7 targeting insulin-like growth factor 1 receptor (IGF-1R), which is an upstream regulator of Akt.

**Methods:**

MicroRNA expression data for gliomas and normal controls were downloaded from The Cancer Genome Atlas (TCGA) database. Quantitative real-time PCR was used to measure the microRNA-7 (miR-7) expression level, and Western blot was performed to detect protein expression in U87 and U251 cells. Colony formation assay and glycolysis stress test were also conducted. Luciferase reporter assay was used to identify the mechanism of IGF-1R and miR-7 regulation.

**Results:**

miR-7 was downregulated in human glioma tissues based on TCGA database. Forced expression of miR-7 or IGF-1R knockdown inhibited colony formation and glucose metabolic capabilities of glioma cells in vitro and decreased the p-Akt expression level. Bioinformatics analysis results indicated that IGF-1R could be a target of miR-7. Western blot and luciferase reporter assays showed that miR-7 modulated IGF-1R expression by directly targeting the binding site within the 3′-untranslated region.

**Conclusions:**

This study provides the first evidence that miR-7 inhibits cellular growth and glucose metabolism in gliomas, at least partially, by regulating the IGF-1R/Akt signaling pathway. Therefore, miR-7 is a promising molecular drug for glioma treatment.

**Virtual Slides:**

The virtual slide(s) for this article can be found here: http://www.diagnosticpathology.diagnomx.eu/vs/13000_2014_211

## Background

Malignant glioma is the most common and lethal primary brain tumor in adults. The fatal nature of malignant gliomas is ascribed to their extensive cell proliferation, intense resistance to cell apoptosis, and widespread infiltration throughout the brain. Despite multimodal therapies, such as surgery, radiotherapy, and chemotherapy, the median survival of glioblastoma multiforme (GBM) is less than one year [1]. Novel therapeutic approaches are required to improve long-term survival for this cancer. Recent advances in our understanding of the biological features of glioma offer opportunities for the design of a new therapeutic strategy based on targeting essential signaling pathways.

Altered energy metabolism is widespread in cancer cells along with other cancer-associated traits that have been accepted as hallmarks of cancer [2]. Otto Warburg first observed an anomalous characteristic of cancer cell energy metabolism. Even in the presence of oxygen, cancer cells can reprogram their glucose metabolism and consequently energy production by limiting their energy metabolism to glycolysis. Such phenomenon is called “aerobic glycolysis.” Increased glycolysis in cancer tissues allows diversion of glycolytic intermediates into various biosynthetic pathways that can synthesize macromolecules and organelles, which are required for assembling new cancer cells [2–4]. Akt may constitute a “Warburg kinase” that can be specifically targeted to alter cancer cell energy metabolism for therapeutic benefits as suggested in previous studies. Akt-induced glycolysis can be mediated by multiple non-exclusive mechanisms, including expression and membrane translocation of glucose transporters. Akt-induced glycolysis can affect hexokinase expression, activity, and mitochondrial interaction. Akt may also indirectly activate the important rate-controlling enzyme phosphofructokinase-1 (PFK1) by directly phosphorylating and activating phosphofructokinase-2 (PFK2) [5]. The principal reaction product of PFK2, i.e., fructose-2, 6-bisphosphate, is the most potent allosteric activator of PFK1. Suppression of glycolytic gene expression by the transcription factor FoxO could be reversed through phosphorylation and inactivation by hyperactive Akt [6]. Akt hyperactivity can increase mTORC1 activity, thereby increasing HIF1α abundance and expression of HIF1α-associated glycolytic enzyme and Glc transporter [7].

Many recent studies demonstrated that dysregulation of a tumor-related microRNA (miRNA) network serves a critical function in glioma development and progression. This network includes miR-181, miR-221/222, miR-21, miR-124, miR-566, and miR-145 [8–14]. Lu also reported that miR-7 exhibits low expression compared with that in normal brain tissues [15]. miR-7 inhibits viability, invasiveness, and metastasis in glioma cells. A previous study showed that some important functional molecules, such as epidermal growth factor receptor (EGFR) and focal adhesion kinase, are the direct target genes of miR-7 [16,17].

In this study, we used miRNA expression data downloaded from The Cancer Genome Atlas (TCGA) database to examine the effects of miR-7 expression. We confirmed that miR-7 served a critical function in cellular growth and metabolism by directly targeting insulin-like growth factor 1 receptor (IGF-1R), which is an upstream regulator of Akt [18,19].

## Methods

### Microarray analysis and cell cultures

MicroRNA expression data for 480 glioma tissues and 10 normal brain tissues were downloaded from TCGA database, and all 480 glioma cases involved GBM (http://cancergenome.nih.gov). All specimens were collected using institutional review board–approved protocols [20]. Human glioma U87 and U251 are two representative cell lines of human GBM; both of these cell lines characterize the consequences of frequent PTEN-null and EGFR overexpression [21]. The U87 and U251 cell lines were purchased from the Chinese Academy of Sciences Cell Bank. All cells were cultured in high-glucose Dulbecco’s modified Eagle’s medium (DMEM; Gibco Corporation, USA) supplemented with 10% fetal calf serum (Hyclone, USA) at 37°C in a humidified atmosphere containing 5% CO^2^. This study was approved by the Research Ethics Committee of Xuzhou Central Hospital, China.

### Cell transfection

All miRNA mimics were chemically synthesized and purified by GenePharma (Shanghai, China) based on the following sequences: has-miR-7 mimic: 5^′^-UGGAAGACUAGUGAUUUUGUUGU-3^′^, miR-negative control (miR-NC):5′-UUCUCCGAACGUGUCCGGAGAATT-3′. IGF-1R and control siRNA oligonucleotide duplexes were chemically synthesized by Invitrogen (si-IGF-1R: sense 5′-CAACAGUGGUCAUCAUGGAACUGAUdTdT-3′, control siRNA (si-NC): sense 5′-UUCUCCGAACGUGUCACGUdTdT-3′). Transfection was performed with Lipofectamine 2000 (Invitrogen) according to the manufacturer’s instructions.

### Quantitative real-time PCR

RNA was extracted from the cells or tissues using TRIzol (Invitrogen). miR-7 (qRT-PCR) reactions were performed using Bulge-loop™ miRNA qRT-PCR Primer (RiboBio, Guangzhou, China) and SYBR Green PCR Master Mix (Applied Biosystems) according to the manufacturer’s protocol. U6 was used for normalization. Relative gene expression was calculated by 2^–ΔΔCt^ method.

### Colony formation assay

Cells were plated onto 35 mm dishes (500 cells/well) in DMEM culture. After 72 h of miR-7 mimic processing, the cells were washed thrice with phosphate-buffered saline (PBS), and fresh broth was supplied. After two weeks, the cells were fixed in 3 mL of 4% paraformaldehyde for 30 min. Giemsa staining was performed for 20 min, and the cells were washed thrice with PBS. The clone number was counted under a microscope.

### Western blot

Cells processed for 48 h were collected, and protein was extracted with lysis buffer containing phenylmethylsulfonyl fluoride. Lysate was centrifuged at 14,000 rpm at 4°C for 15 min, and protein content was measured using bicinchoninic acid method. Up to 40 μg of proteins was separated using 10% SDS–PAGE and transferred to polyvinylidene difluoride membrane. The membrane was incubated with primary antibodies overnight at 4°C and with secondary antibodies for 2 h at 25°C. The following antibodies were used: IGF-1R (1:1000, CST, USA), p-Akt (1:1000, CST, USA), and Akt (1:1000, CST, USA). β-actin (1:1000, CST, USA) was used as an internal protein control.

### Luciferase reporter assays

IGF-1R 3′-untranslated region (UTR)-Luc reporter assay was performed by ligating the IGF-1R 3′-UTR PCR product into the XbaI site of the pGL3 control vector (Invitrogen). The mutant-type reporter was generated by deleting the binding site of miR-7 “GUCUUCC.” The cells were co-transfected with wild-type (pGL3-WT-IGF-1R-3′-UTR) or mutant-type (pGL3-MUT-IGF-1R-3′-UTR) luciferase reports and miR-7 mimic or miR-NC. After 48 h of incubation, luciferase activity was measured using the Dual Luciferase Reporter Assay System (Promega, Madison, USA).

### Glycolysis stress test

Glycolysis and glycolytic capacities were determined for U87 and U251 cells using the Seahorse Extracellular Flux (XF-96) analyzer (Seahorse Bioscience, Billerica, MA) [22]. Cells were seeded in XF96-well plates and incubated at 37°C in a 5% CO_2_ humidified atmosphere for 24 h. Extracellular acidification rates (ECARs) were simultaneously measured real time after 1 d in XF-96. Initially, the cells were incubated in the glycolysis stress test medium without glucose, and ECARs were assessed. Three sequential injections of D-glucose (10 mM), oligomycin (1 μM), and 2-deoxyglucose (100 mM) were injected in turn, and ECARs were assessed. Non-glycolytic acidification was defined as initial and final ECARs. Glycolysis was defined as ECAR following addition of D-glucose and maximum glycolytic capacity, which was defined as ECAR following addition of oligomycin.

### Statistical analysis

All experiments were performed in triplicate. Data were expressed as mean ± SD. ANOVA and Student’s *t-*test, which were based on SPSS 16.0 software, were used to analyze the statistical differences. A two-sided *P* <0.05 was considered statistically significant.

## Results

### miR-7 expression and function in glioma cells in vitro

**Figure 1:**
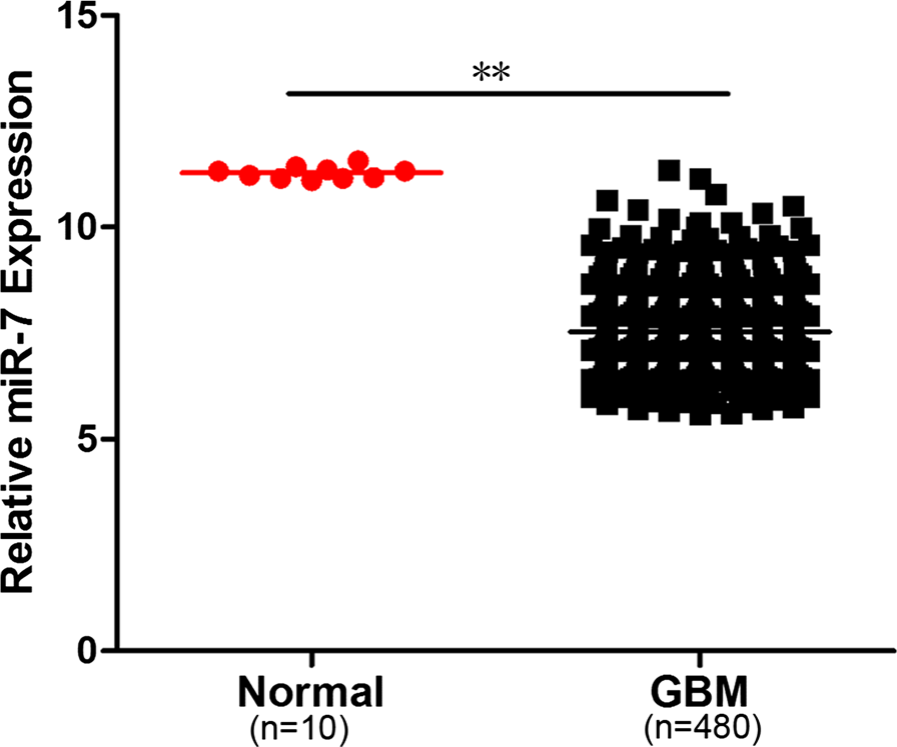
**Clinical significance of miR-7 in glioma cases and normal brain tissues.** AveragemiR-7 expression in glioma cases (n =480) and normal (n =10) tissues by microarray. ^**^p <0.01.

**Figure 2:**
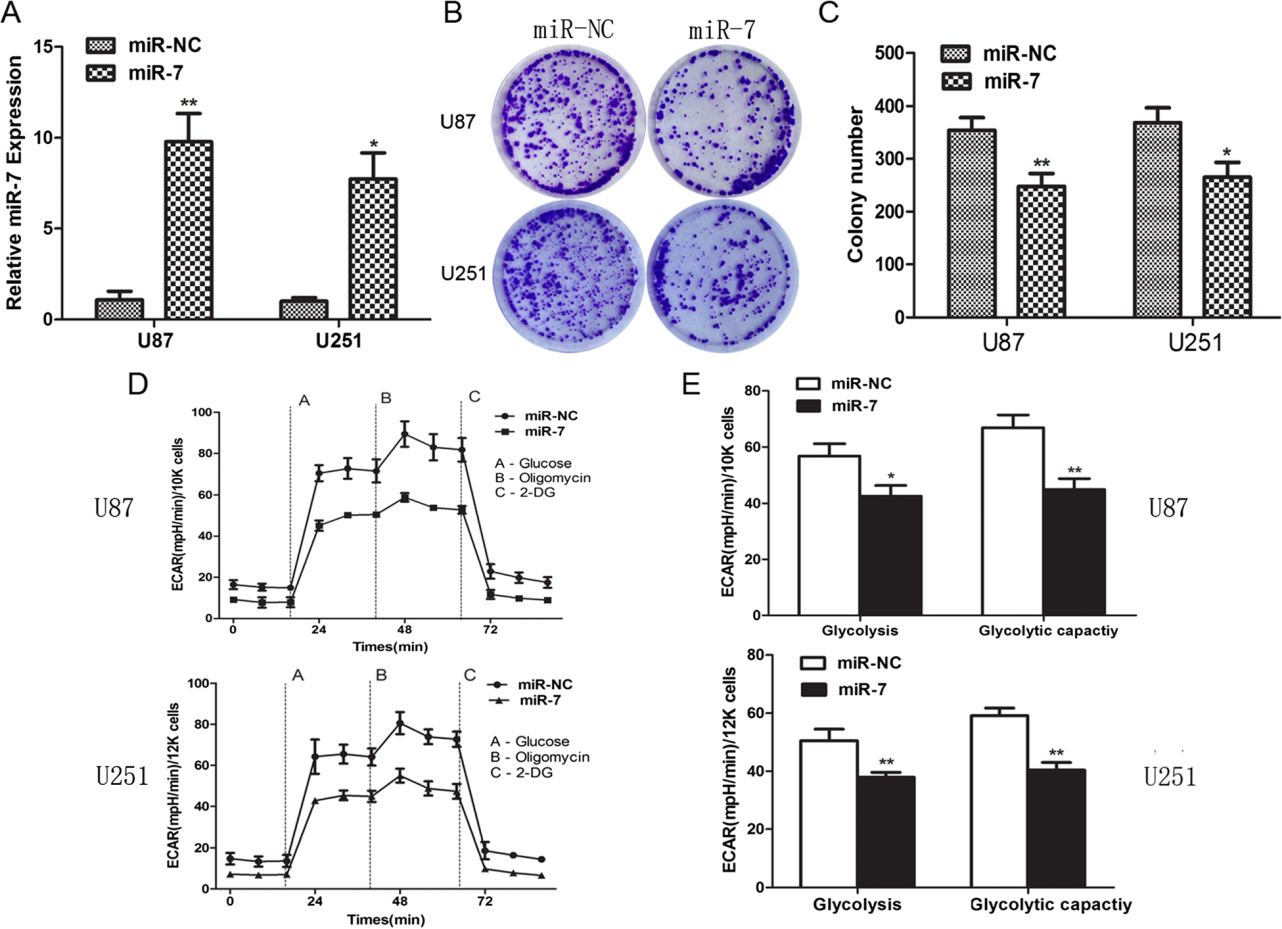
**miR-7 suppresses glioma cellsgrowth and glycometabolismin vitro. (A)** The expression levels of miR-7 were determined by qRT-PCR in bothU87 cells and U251 cells. **(B, C)** Colony formation assay was used to detect the colony formation activity. **(D, E)** The glycolytic activity and maximum glycolytic capacity was determined in real-time using the Seahorse extracellular flux analyzer. ECAR were continuously calculated for two hours. ECAR following the addition of glucose defines glycolysis and ECAR following oligomycin represents maximum glycolytic capacity. Each data point represents the mean ± SD of three experiments. ^*^p <0.05, ^**^p <0.01.

In TCGA database, the miR-7 expression in the GBM group was significantly lower than that in the normal brain tissue group (Figure 1). To identify the effects of miR-7 on glioma cells, we conducted the following functional assays. First, the miR-7 expression level in the cells transfected with miR-7 mimics was determined by real-time PCR (Figure 2A). Cell tablet assays revealed that miR-7 overexpression can significantly inhibit the number of colonies (Figure 2B and C). To assess the function of miR-7 in glucose metabolism, we performed a glycolysis stress test. Upregulation of miR-7 weakened the glycolysis and glycolytic abilities of glioma cells compared with those of the control (Figure 2D and E).

### IGF-1R as a direct target of miR-7

**Figure 3:**
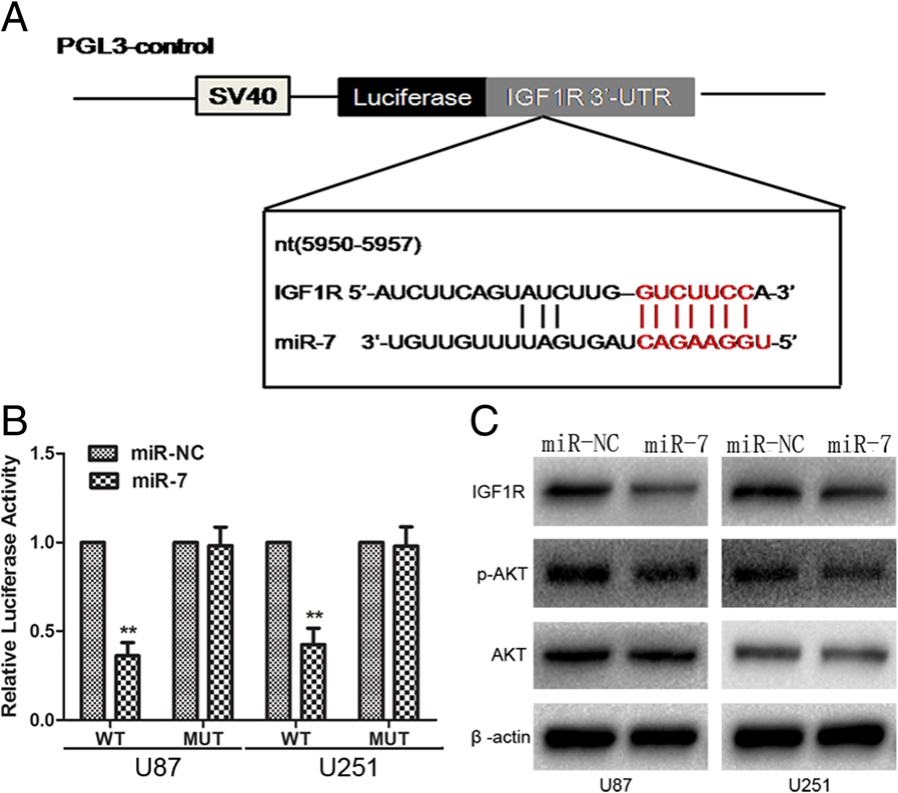
**IGF-1R is a direct target of miR-7 in glioma cells. (A)** Diagram of seed sequence of miR-7 matched the 3′UTR of the IGF-1R gene. **(B)** Luciferase reporter assays in glioma cells, following co-transfection of cells with wild-type or mutant 3′UTR IGF1R and miRNA, as indicated. **(C)** Effect of miR-7 on IGF-1R, AKT, pAKT protein levels. Each data point represents the mean ± SD of three experiments. ^**^p <0.01.

To further clarify the molecular mechanisms of miR-7 in tumor suppression, we used a target prediction program, TargetScan, to predict the putative targets of miR-7. The 3^′^-UTR of IGF-1R mRNA contained a complementary site for miR-7 (Figure 3A). Luciferase activity assays were conducted to confirm whether IGF-1R is a putative target of miR-7. The wild- or mutant-type luciferase reporter plasmids were constructed and co-transfected with miR-7 mimics or scrambled into glioma cells. Reporter assay results revealed that miR-7 overexpression led to a significant decrease in the luciferase activity of pGL3-WT-IGF-1R without changing that of pGL3-MUT-IGF-1R 3^′^-UTR (Figure 3B). Accordingly, Western blot analysis showed that the levels of IGF-1R and its downstream molecular events decreased after transfection of miR-7 compared with those in the miR-NC group (Figure 3B). Thus, miR-7 could directly regulate the IGF-1R/Akt signaling pathway in glioma cells.

### IGF-1R downregulation inhibits glioma cell growth and glycometabolism in vitro

**Figure 4:**
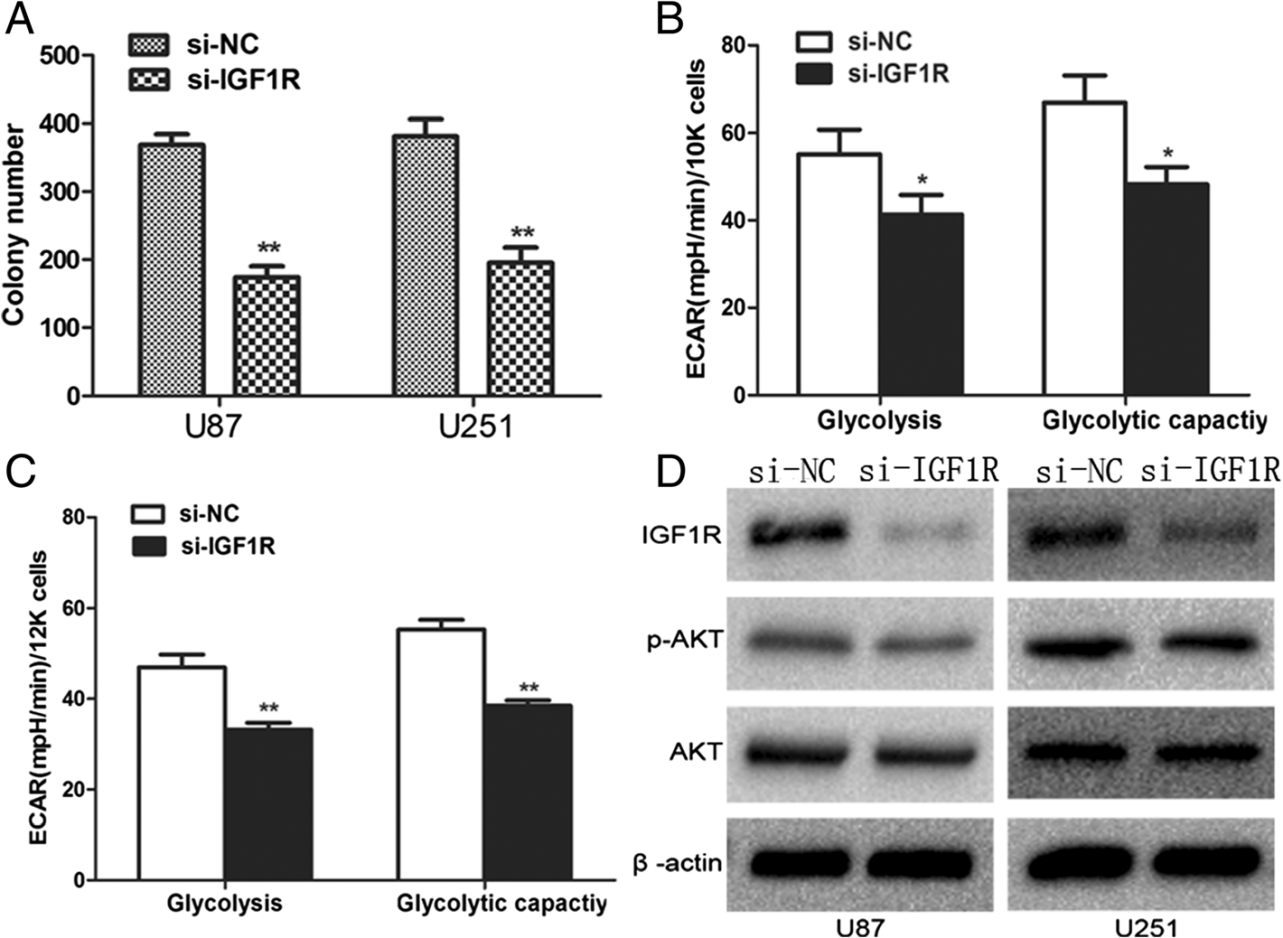
**IGF1R impact growth and glycometabolism of glioma cells. (D)** IGF-1R, AKT, pAKT protein levels in U87 cells and U251 cells transfected with siIGF1R were assessed by Western blot. **(A, B, C)**. Representative cartogram showing proliferation and glycometabolism regulated by siIGF-1R. Each data point represents the mean ± SD of three experiments. ^*^p <0.05, ^**^p <0.01.

We performed the following functional assays to explore the function of IGF-1R in cellular growth and metabolism. Western blot was used to identify the downregulation of IGF-1R by siRNA (Figure 4D). Similar to the treatment with miR-7, the ability of colony formation and glucose metabolism decreased after IGF-1R inhibition (Figure 4A, B, and C). As expected, the activity of AKT signaling was suppressed by si-IGF-1R (Figure 4D).

## Discussion

Recent studies have focused on molecular factors, which serve a function in carcinoma development. Thus, as a prospective consequence, novel treatment strategies targeting these factors and their receptors have been improved. The IGF signaling axis is among the major target themes of many studies searching for new strategies in tumor treatment. The IGF signaling axis comprises three growth factors (IGF-1, IGF-2, and insulin), three membrane receptors (IGF-1R, IGF-2R, and IR), six circulating IGF-binding proteins (IGFBP1 to IGFBP6), and proteases that modulate ligand availability [23,24]. IGF-1R serves many important functions in various pathways of mitogenesis, angiogenesis, transformation, apoptosis, and cell motility [25]. IGF-1Rs also interfere with mitogenic and antiapoptotic events in malignant cells. Thus, IGF-1R serves a potential function in carcinogenesis. Activated phosphorylated IGF-1R recruits and activates signaling adaptor proteins, including IRS-I, IRS-2, and Shc [26]. IRS phosphorylation activates the phosphoinositide-3-kinase/Akt pathway, thereby resulting in the synthesis of membrane-associated phosphatidylinositol (3, 4, 5)-trisphosphate. Consequently, Akt and protein kinase B are activated. Akt is a kinase-activating molecule that induces antiapoptotic proteins [27]. As a result of this signaling, many IGF-1R effects are mediated, including mitogenesis, proliferation, cell-cycle control, and inhibition of apoptosis [28]. We examined the mechanisms underlying the loss of IGF-1R-inhibited cellular growth and metabolism through the Akt pathway in glioma cells. Downregulation of IGF-1R inhibited the activity of Akt and suppressed cellular growth and metabolism.

Dysregulation of miRNA sequences is a common feature in human cancers, including glioma. miRNA is a small non-coding single-stranded RNA comprising 21 nucleotides to 25 nucleotides and regulates the expression of target genes by interacting with specific sites on messenger RNA, thereby repressing protein translation. miRNA sequences have important regulatory functions in basic biological processes, such as development, cellular differentiation, proliferation, and apoptosis. Altered miRNA regulation is involved in glioma pathogenesis via oncogene and tumor suppressor modulation, which subsequently affects downstream signaling pathways [29–31]. Consistent with previous reports, miR-7 was downregulated in human glioma tissues in the current study [17]. Upregulation of miR-7 inhibited cellular growth and glucose metabolism. Bioinformatics analysis results indicated that IGF-1R could be a target of miR-7. Western blot and luciferase reporter assays showed that miR-7 modulated IGF-1R expression by directly targeting the binding site within the 3′-UTR.

## Conclusion

This study provides the first evidence that miR-7, as a regulator of AKT pathway, serves a critical function in cellular growth and glucose metabolism by directly targeting IGF-1R. Therefore, miR-7 is a promising molecular drug for glioma treatment.
